# Efficiency of a novel thermosensitive enema *in situ* hydrogel carrying *Periplaneta americana* extracts for the treatment of ulcerative colitis

**DOI:** 10.3389/fphar.2023.1111267

**Published:** 2023-02-08

**Authors:** Ming Wu, Hui Ding, Xiao Tang, Jiayi Chen, Meng Zhang, Ziqiong Yang, Qian Du, Jun Wang

**Affiliations:** ^1^ Institute of Pediatrics, Xuzhou Medical University, Xuzhou, China; ^2^ Department of Pediatrics, Xuzhou Medical University Affiliated Hospital, Xuzhou, China; ^3^ School of Pharmacy, Xuzhou Medical University, Xuzhou, China; ^4^ Jiangsu Key Laboratory of New Drug Research and Clinical Pharmacy, Xuzhou Medical University, Xuzhou, China

**Keywords:** *Periplaneta americana*, thermosensitive *in situ* gel, ulcerative colitis, rectal delivery, necroptosis

## Abstract

**Objective:** The aim of this study was to develop a thermosensitive *in situ* gel (TISG) as an effective rectal delivery platform for delivering *Periplaneta americana* extracts (PA) to alleviate ulcerative colitis (UC) and explore the underlying molecular mechanism.

**Materials and methods:** Thermosensitive (poloxamer 407) and adhesive polymers (chondroitin sulfate modified carboxymethyl chitosan, CCMTS) were used to construct the *in situ* gel. CCMTS and aldehyde poloxamer 407 (P407-CHO) were synthesized and chemically cross-linked by Schiff base reaction to formulate thermosensitive *in situ* gel, which carried *Periplaneta americana* extracts (PA/CCMTS-P). The cytotoxicity and cellular uptake of CCMTS-P were investigated in lipopolysaccharide (LPS) -induced macrophages by CCK-8 assay. The anti-inflammatory effects of PA/CCMTS-P were studied in lipopolysaccharide-induced RAW264.7 cells and dextran sulfate sodium (DSS)-induced ulcerative colitis mouse models. In addition, the ability of PA/CCMTS-P to restore the intestinal mucosal barrier after rectal administration was evaluated by immunohistochemical analysis (IHC).

**Results:** PA/CCMTS-P was prepared and characterized as gel with a phase-transition temperature of 32.9°C. The results of the *in vitro* experiments indicated that the hydrogels promoted the cellular uptake of *Periplaneta americana* extracts without causing any toxicity as compared to the free gel. PA/CCMTS-P showed superior anti-inflammatory activity both *in vitro* and *in vivo,* which restored the damaged intestinal mucosal barrier associated by inhibiting necroptosis in dextran sulfate sodium-induced ulcerative colitis models.

**Conclusion:** The findings from our study show that the rectal administration of PA/CCMTS-P holds a promising potential for the treatment of ulcerative colitis.

## Introduction

Ulcerative colitis (UC) is a chronic inflammatory disease that is characterized by abnormal immune function, accompanied by mucosal barrier damage and infiltration of inflammatory factors (Ng et al., 2017). The clinical manifestations of UC include diarrhea, abdominal pain, weight loss, mucopurulent bloody stools and some complications such as colon cancer and extraintestinal manifestations (Feuerstein and Cheifetz, 2014). Despite the currently available treatments such as amino-salicylic acids, corticosteroids, immunomodulators, monoclonal antibodies and fecal microbiota transplantation, management of UC is still a global challenge due to its complex etiology, variable complications and uncertain therapeutic outcomes.

At present, natural anti-inflammatory traditional Chinese medicines have garnered wide attention as alternative therapies for UC, especially for patients who do not respond to the standard treatments. *Periplaneta americana* dried insects is a traditional Chinese medicine and pharmacological studies have demonstrated its ability to promote blood circulation, diuresis, and wound healing. Kangfuxin liquid (KFX) is an NMPA-appoved Chinese patented medicine composed solely of the extracts of *Periplaneta americana* dried insects (PA), with the function of promoting blood circulation and nourishing yin and promoting granulation (Xue NN et al., 2020). Recent studies have reported the therapeutic effects of PA on UC, which suppressed the release of pro-inflammatory factors, restored intestinal barrier function, regulated the dysregulated gut microbiota, thereby restoring the balance in the intestinal-immune system (Ma et al., 2018; Xue NN et al., 2020; Ni et al., 2022).

Macrophages play an important role in regulating immune response both in innate and acquired immunity (Biswas and Mantovani, 2010). They essentially contribute to chronic inflammatory diseases due to their strong plasticity. Previous studies have shown that macrophages can effectively modulate the intestinal immune system and regulate the inflammatory microenvironment, thereby reducing the inflammatory response associated with UC (Xiao et al., 2017; Zhang et al., 2020). Meanwhile, the highly expressed CD44 receptor on macrophages provides a potential binding site for active targeting by anti-UC medicines (AbdElazeem and El-Sayed, 2015; Gou et al., 2019).

Necroptosis is a type of programmed cell death, and receptor-interacting protein kinase 1 (RIP1), receptor-interacting protein 3 (RIP3) and mixed lineage kinase domain-like protein (MLKL) are critical kinases involved in the necroptosis pathway. Meanwhile, RIP1/RIP3/MLKL mediated necroptosis acts as a trigger for necroinflammation, which triggers downstream inflammatory responses by activating the NOD-like receptor family pyrin domain containing 3 (NLRP3) inflammasome (Wang X et al., 2014). Cysteine protease-1 (Caspase-1) released by NLRP3 inflammasomes further stimulates the release of pro-inflammatory factors such as IL-1β (Interleukin-1β) and IL-18 (Interleukin-18), which aggravate inflammation. It was shown that RIP3 and MLKL were upregulated in the inflamed tissues of UC patients, and were positively correlated with the severity of UC (Wu et al., 2019). The RIP1/RIP3/NLRP3 signaling pathway mediated necroinflammation is known to be involved in UC (Wu et al., 2021).

In the last few years, novel drug delivery systems have been increasingly explored for treating UC, and have mainly focused on its pathological condition. Despite the poor rate of patient acceptability limiting its widespread use, rectal administration of medicinal products constitutes a longstanding practice. Cases of disease localized at the terminal gastrointestinal tract are particularly suitable for rectal drug administration. The rectal route has many advantages, including low susceptibility to enzymatic degradation and ability to bypass the hepatic first-pass effect, thus providing a relatively static environment for the absorption of drugs (Jannin et al., 2014). However, enemas often cause anal leakage leading to inadequate dosing when administered in liquid preparation. While solid suppositories are often accompanied by discomfort to the patient. Therefore, thermosensitive *in situ* gels (TISG) which can transform from a viscous liquid state to gel *in situ* upon exposure to changing temperature, have been considered as potential alternatives for alleviating these limitations. The development of TISG has prompted investigation of many drugs such as curcuminoids, artesunate (Gaudin et al., 2008), and 5-fluorouracil (Wang et al., 2020) to increase drug bioavailability.

Hence, we developed an effective TISG formulation containing PA to treat UC, which may be easy to administer, potentially enhance the regional delivery of PA, thereby reducing the dosing frequency of PA. Various materials have been proposed and used for producing TISG. Herein, poloxamer 407 was chosen due to its extensive safety record, low cost, and approval by major medicines regulatory agencies including the FDA and EMA (Mommerot et al., 2007). Moreover, chitosan (CS) and its hydrosoluble derivatives carboxymethyl chitosan (CMCS) are also highly suitable materials for rectal preparations owing to their properties such as biocompatibility, biodegradability, antimicrobial activity and mucoadhesivity. It was reported that cross-linked hydrogels had stronger mucosal adhesion and gelling strength than the gels consisting of single P407 or chitosan (Meng et al., 2022). In this work, we designed TISG formulation composed of P407 cross-linked chondroitin sulfate modified CMCS (CCMTS) for the rectal delivery of PA. The hydrogel was further characterized by testing on a mouse model through *in vivo* and *in vitro* imaging. Overall, the objective of the present study was to formulate a novel thermosensitive *in situ* gel system loaded with PA and evaluate its effect on regulating necroptosis in DSS-induced mouse model of UC.

## Materials and methods

### Reagents and antibodies

PA stock solution was a gift from Good Doctor Pharmaceutical Group (Sichuan, China). P407 was purchased from H and C Fine Chemical (Shanghai, China) was purchased from BASF SE (Ludwigshafen, German). *e*-Poly-L-lysine (ε-PL) was purchased from Aladdin (Shanghai, China) and CMCS was procured from Yuanye Bio-Technology (Shanghai, China). Chondroitin sulfate (CS), Rhodamine B (RB) and Fluorescein isothiocyanate (FITC) fluorochrome were purchased from Solarbio (Beijing, China). DSS (MW 36–50 kDa) was purchased from MP Biomedicals (Santa Ana, CA, United States). LPS (*E. coli* serotype O55:B5) was purchased from Sigma-Aldrich (Missouri, United States). Mouse IL-6 (Interleukin-6), IL-1β, IL-18, and TNFα (Tumor necrosis factor α) ELISA (enzyme-linked immunosorbent assay) kits were purchased from Elabscience Biotechnology (Wuhan, China).

Primary antibodies for Zonula occludens-1 (ZO-1, ab96587), Claudin-3 (ab15102) and Occludin (ab216327) were procured from Abcam (Cambridge, UK). Primary antibodies for E-Cadherin (#3195), RIP1 (#3493), RIP3 (#13526), Phospho-RIP3 (#91702), MLKL (#14993), Phospho-MLKL (#37333), NF-κB p65 (#4764) and F4/80 (#30325) were purchased from cell signaling technology (Danvers, MA, United States). Primary antibody against NLRP3 (NBP2-12446) was purchased from Novus Biologicals (Littleton, Colorado, United States), Caspase-1 (p20) (AG-20B-0042-C100) was purchased from Adipogen (San Diego, United States) and beta-actin (AF7018) was purchased from Affinity (Melbourne, Australia). Anti-mouse (abs20001) and anti-rabbit (abs20002) IgG conjugated with horseradish peroxidase (HRP) were purchased from Absin (Shanghai, China) and CoraLite594 conjugated goat anti-rabbit IgG (SA00013-4) was purchased from Proteintech (Wuhan, China).

CCK8 assay kit was procured from Med Chem Express (New Jersey, United States). Immunohistochemical (IHC) staining kit was purchased from ZSJQ-BIO (Beijing, China). Hematoxylin and eosin (H&E) staining kit, DAPI and bicinchoninic acid (BCA) protein kit were purchased from Beyotime Biotech (Shanghai, China).

### Synthesis and structural identification of CCMTS, P407-CHO

CCMTS: 53 mg CMCS was weighed and added into a 50 mL round-bottom flask, 10 mL purified water was added and dissolved. 25 mg N-hydroxysuccinimide and 21 mg 1-ethyl- (3-dimethylaminopropyl) carbonyl diimide hydrochloride were added and reacted for 2 h in dark. After adjusting the pH to 7.5 with 0.1 mol/L sodium hydroxide solution, 30 mg CS was added and the reaction was allowed to continue for 24 h. After pure water dialysis for 24 h (MW3500), the reaction was freeze-dried to obtain the polymer, chondroitin sulfate modified carboxymethyl chitosan (CCMTS). Certain amount of CCMTS, CMCS, and CS were dissolved in heavy water (D_2_O) for ^1^H NMR analysis (AVANCE Ⅲ, German).

P407-CHO: 100 mg P407, 366 mg p-aldehyde benzoic acid, 629 mg dicyclohexyl carbimide and 29.50 mg 4-dimethylaminopyridine were weighed, and then successively added to 50 mL double-necked round-bottom flask. Then 9.5 mL tetrahydrofuran was added and the reaction was stirred under nitrogen protection for 12 h. The filtrate of the reaction solution was dispersed in diethyl ether, and the precipitate was obtained by centrifugation. After repeating the above steps for three times, the precipitate was dried at 40°C to obtain aldehyde P407 (P407-CHO). The product was pressed with potassium bromide and analyzed by FT-IR (SHIMADZU, Japan). Certain amount of P407-CHO and P407 were dissolved in dimethyl sulfoxide for analysis by ^1^HNMR.

### Preparation and characterization of PA/CCMTS-P

Preparation: CCMTS, P407-CHO, and P407 were slowly added to purified water under stirring. They were allowed to stand until full swelling in the refrigerator at 4°C for 12 h. Then P188 solution, *ε*-PL solution and PA concentrate were added and PA/CCMTS-P was made by stirring. Among them, the mass concentration of P407 was 21%, P188 was 0.2%, and polylysine was 0.4%. The concentration of PA in PA/CCMTS-P was consistent with its concentration in the patented Chinese medicine Kangfuxin liquid.

Phase-transition temperature determination: 10 g PA/CCMTS-P TISG and a stirrer were put into a vial, and a precision thermometer with an accuracy of 0.1°C was inserted into it. The mercury sphere of the thermometer was completely immersed in the gel solution. The gel was placed at a low temperature (<10°C) water bath with a rotating speed of 300 rpm/min, and the water temperature raised continuously and slowly about 1°C–2°C/min. The temperature at which the stirrer stopped rotating completely was set as the gelation temperature, and the measured time was averaged after repeating the above procedure thrice.

Rheological characterization: At 37°C, the gelled PA/CCMTS-P was placed on the measuring mold of the rotary rheometer (Anton Paar, Austria). Firstly, the amplitude of the PA/CCMTS-P was scanned to determine the linear viscoelastic region, that is, the storage modulus and loss modulus of the sample were independent of the applied strain. After that, the storage modulus (G′) and loss modulus (G″) at different angular frequencies were tested at 37°C and in the measured linear viscoelastic region with a frequency range of 0.1–100 rad/s.

### Cell culture and cell viability assay

Raw264.7 cells were obtained from Cell Center of Chinese Academy of Sciences (Shanghai, China) and cultured in dulbecco’s modified eagle medium (DMEM, KeyGEN, Jiangsu, China) containing 20% fetal calf serum (Gibco, California, United States), 1% penicillin/streptomycin sulfate (Beyotime, Beijing, China) at 37°C in a humidified 5% CO_2_ atmosphere.

Cell viability was assessed by the CCK8 assay. Raw264.7 cells were plated into 96-well plates (1 × 10^4^ cells/well) and stimulated with the PA stock solution at different concentrations (0.05, 0.1, 0.2, 0.4 mg/mL), with different dilutions of CCMTS-P (1:225, 1:450, 1:900, 1:1800) and at different concentrations of PA/CCMTS-P (containing PA stock solution 0.05, 0.1, 0.2, 0.4 mg/mL) for 24 h. Meanwhile, different concentrations of LPS (0, 25, 50, 100, 200, 400 ng/mL) were added to Raw264.7 cells for 24 h to identify the optimal stimulus concentration. 10μL CCK-8 solution was then added to each well. After incubating at 37°C for 1–2 h, the optical density (OD) values were measured at 450 nm using a scanning multi-well spectrophotometer (Biotek, United States).

### *In vitro* cellular uptake

Quantitative and qualitative cellular uptake was measured by flow cytometry (Agilent, United States) and fluorescent microscope (Olympus, Japan). Raw264.7 mouse macrophage cells were seeded into 12-well plates (1 × 10^6^ cells/well). After stimulation with LPS (100 ng/mL) for 24 h, fresh DMEM containing 450 times diluted CCMTS and 5 μg/mL RB were added. For the blank group, DMEM containing 5 μg/mL RB alone was added for 1 h. After incubation for 1, 2, and 4 h, the cells were washed twice with PBS, gently dissociated with plastic pipette, centrifuged at 1,500 g for 5 min, resuspended in 0.2 mL of PBS, and analyzed by flow cytometry. Furthermore, fluorescent microscope was used to observe the distribution of RB labeled CCMTS in Raw264.7 cells after incubation with RB@CCMTS for 1, 2, and 4 h. After washing twice with PBS, the cells were fixed with 4% paraformaldehyde for 10 min and treated with DAPI for staining the nuclei. The images were collected by fluorescent microscope at ×200 magnification.

### Experimental animals and treatment

Female C57BL/6 mice (weight 18–21 g) were obtained and housed in the Medical Experimental Animal Centre of Xuzhou Medical University. All experiments were approved by the Ethics Committee of Xuzhou Medical University. The mice were randomly divided into six groups (6 mice per group), including NS, Model, PA (ig), CCMTS-P, PA/CCMTS-P (low dose) and PA/CCMTS-P (high dose) group. The NS group received no treatment, while the remaining five groups had free access to drink 3% DSS for 7 days before reverting to normal drinking water. On day 8, mice in the PA (i.g.) group were gavaged with 1:10 times diluted PA stock solution (5 μL/g per day). For the CCMTS-P group, 5 μL/g CCMTS-P was administered by enema per day. PA/CCMTS-P (low dose) and PA/CCMTS-P (high dose) groups were given 5 μL/g CCMTS-P containing PA stock solution 0.09 g/mL and 0.18 g/mL, respectively, by enema every day. The NS and Model groups were given normal saline (5 μL/g) every day. The body weights were monitored daily on fixed time. On day 12, all mice were sacrificed under anesthesia, and the colon tissue was measured and collected.

### Permeation effect *in vivo*

To further study the targeted permeability of CS-functionalized CCMTS-P into macrophages *in vivo*, the fluorescent probe FITC and F4/80 were used to label CMTS-P, CCMTS-P and macrophages, respectively. Then, 3% DSS-induced UC mice were injected with FITC@CMTS-P (no CS) or FITC@CCMTS-P (linking CS) through rectal enema administration. 2 h after enema administration, mice were sacrificed and their colon tissues were extracted, rinsed thoroughly with PBS, and embedded in optimal cutting temperature (OCT) compound. Colon sections (20 μm) were stained with F4/80 and DAPI. Images of the tissue sections were acquired by fluorescent microscope (Olympus, Japan).

### Histological examination

The harvested colon tissues were fixed in 4% paraformaldehyde, embedded in paraffin and then cut into 3 μm sections. The tissues were stained with hematoxylin and eosin (H&E). Degree of inflammation and mucosal damage were assessed by Olympus microscope (Olympus, Japan) at ×40 and ×200 magnification in a blinded fashion.

### Inflammatory cytokines test

Raw264.7 cells were seeded into 24-well plates (5 × 10^5^ cells/well), LPS (100 ng/mL) was added with PA (0.2 mg/mL) or PA/CCMTS-P (containing PA 0.2 mg/mL) for 24 h. Following stimulations, the supernatants were collected and stored at −80°C until further analysis. The expression of inflammatory factors including TNF-α, IL-6, IL-1β and IL-18, were measured in the supernatants using ELISA kits according to the manufacturer’s instructions. Similarly, the levels of IL-1β, and IL-18 in the colon tissue homogenate were measured based on the manufacturer’s instructions for the ELISA kits. Concentration of the cytokines were calculated using standard curves.

### Western blotting assay

Total protein was extracted from the colon tissues after homogenizing and lysing the samples in matrix tubes (MP Biomedicals). The protein concentration was determined using the BCA protein kit. Equal amounts of protein extracts were separated on 10% SDS-polyacrylamide gels and trans-blotted onto polyvinylidene difluoride (PVDF) membranes. The membranes were then blocked with skimmed milk at room temperature for 1 h and incubated overnight at 4°C with the respective primary antibodies (1:1,000 dilution). The next day, the membranes were incubated with the secondary antibody (1:5,000 dilution) for 2 h at room temperature. Finally, after washing in the TBS-T washing solution three times, the protein bands were visualized with an enhanced chemiluminescence detection system (Bio-rad, United States). The protein expression in each sample was normalized with respect to the loading control beta-actin, with the software ImageJ 1.47V (National Institutes of Health, United States).

### Immunohistochemical analysis

Serial sections of embedded colon tissue from each group were used for IHC analysis by using IHC staining kit. After being deparaffinized, hydrated, and heated in citric acid buffer at 95°C for 15 min, the slides were blocked with 5% bovine serum albumin (BSA) for 30 min at 37°C and incubated overnight at 4°C with primary antibodies against ZO-1 (1:300), Claudin-3 (1:100), Occludin (1:200) and E-cadherin (1:400), respectively. Then the sections were incubated with the respective secondary antibodies at room temperature for 1 h before being visualized with diaminobenzidine. Images were assessed by Olympus microscopes (Olympus, Japan) at ×40 and ×200 magnification in a blinded fashion.

### Statistical analyses

All data were analyzed using SPSS 23.0 software (IBM SPSS Statistics, NY, United States). The data are presented as the mean ± standard deviation. One-way analysis of variance was performed among the groups and *p <* 0.05 was considered to be statistically significantly different.

## Results

### Structural characterization of P407-CHO and CCMTS

**FIGURE 1:**
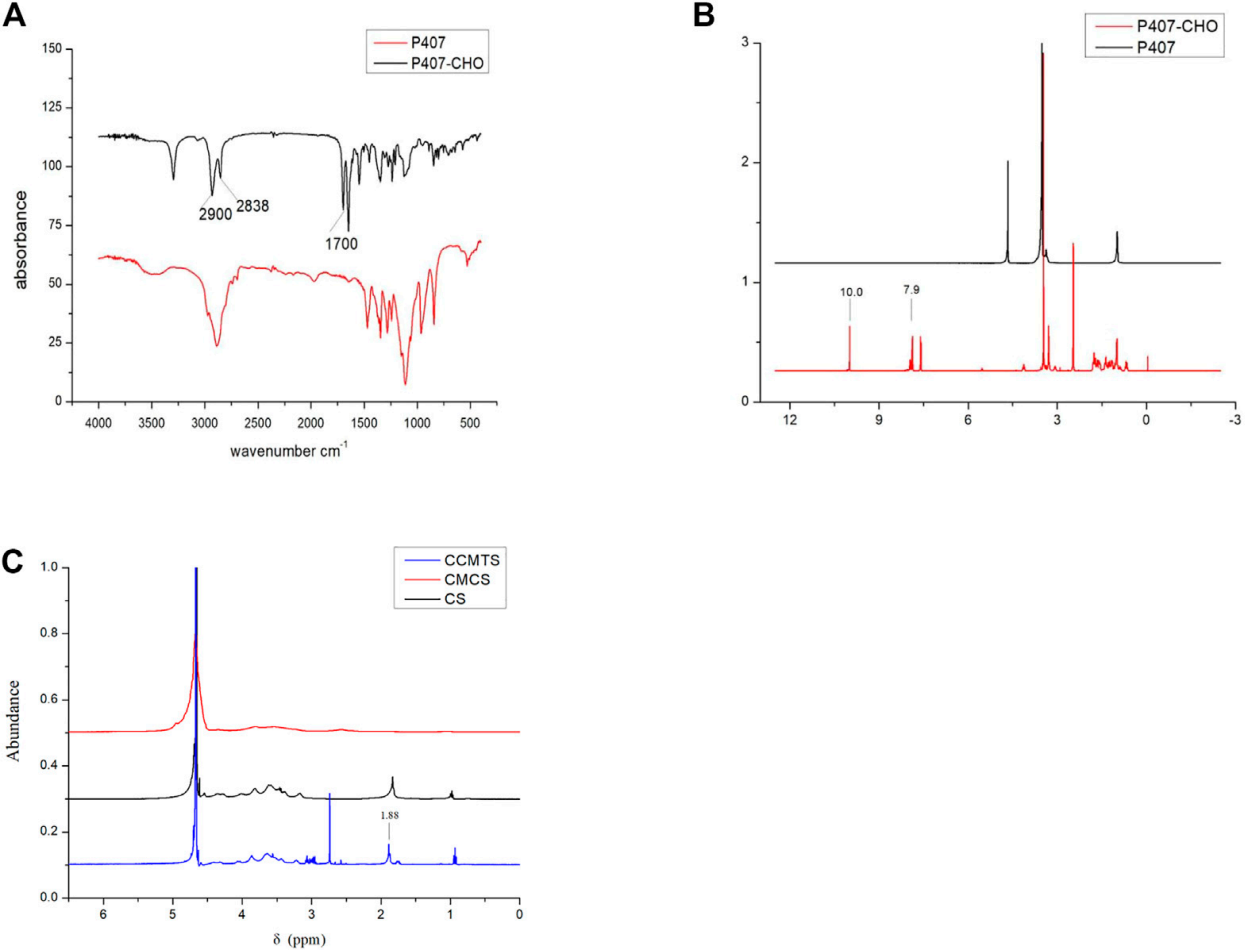
Structural analysis of P407-CHO and CCMTS. **(A)** FT-IR spectrum of P407 and P407-CHO. **(B)** ^1^H NMR images of P407 and P407-CHO. **(C)** ^1^H NMR images of CCMTS, CMCS, and CS.

FT-IR profiles of P407 and P407-CHO are shown in Figure 1A. The C-H stretching vibration peaks of aldehyde group were located at 2,900, 2,838, and 1700 cm^−1^ corresponded to the stretching vibration peak of the aldehyde carbonyl group, which suggested that the aldehyde group was successfully connected to P407 through reaction. As shown in Figure 1B, the proton peak of the benzene ring was linked to the aldehyde group at δ = 7.9 ppm, and δ = 10.0 ppm corresponded to the proton peak of the aldehyde group, which demonstrated the successful synthesis of P407-CHO. As shown in Figure 1C, δ = 1.88 ppm was the methyl proton peak linked to the double bond, which indicated the successful grafting of CS to CMCS and the successful synthesis of CCMTS.

### Preparation and characterization of PA/CCMTS-P

The phase change temperature of PA/CCMTS-P was determined as (32.9 ± 0.4) °C (*n* = 3), higher than room temperature and slightly lower than the rectal temperature, which was suitable for enema administration.

**FIGURE 2:**
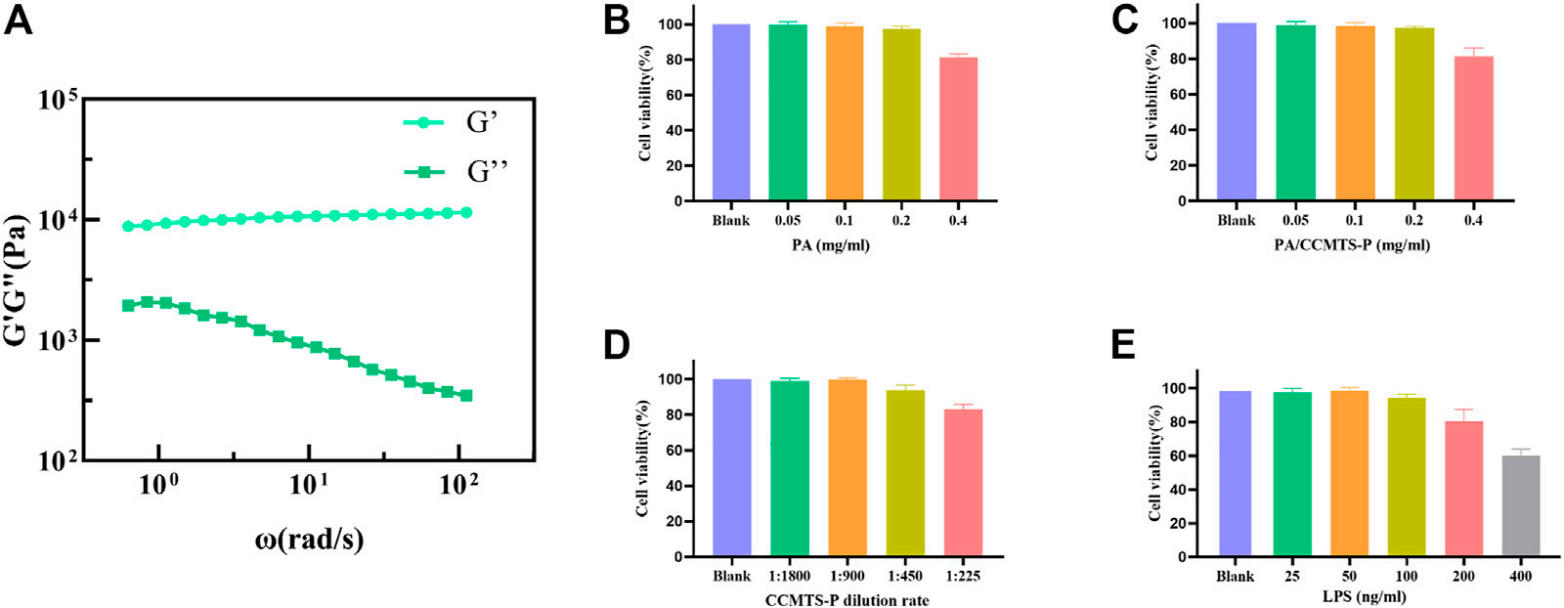
Characterization of PA/CCMTS-P and the results of cell viability assay. **(A)** Rheological property of PA/CCMTS-P, and G′ represents the elastic property while G″ represents the viscous property. **(B–D)** cytotoxicity of the PA stock solution, CCMTS-P and PA/CCMTS-P at different concentrations to Raw264.7 cells at 24 h. **(E)** The cytotoxicity of LPS to Raw264.7 cells after 24 h stimulation.

Rheological property analysis: The hydrogel is subjected to cyclic shear stress from body fluids in the patients’ body. We tested the stability of the gel under cyclic stress by exposing to angular frequency scanning of the rheometer. The storage modulus (G′) and loss modulus (G″) of PA/CCMTS-P were tested, where G′ represented the elastic property and G″ represented the viscous property of the hydrogel. As shown in Figure 2A, in the whole frequency scanning range, G′ curve was always higher than the G″ curve, which showed that the hydrogel was obviously dominated by elastic properties. Besides, G′ was relatively stable without significant frequency dependence, indicating that the gel always had a stable three-dimensional network structure.

### Analysis of the cell viability

As shown in Figure 2E, LPS was used to stimulate Raw264.7 cells to establish an inflammation model *in vitro* (He and Frost, 2016). It was found that 25 and 50 ng/mL LPS did not affect the viability of Raw264.7 cells after 24 h treatment, and the cell viability decreased to nearly 90% at the concentration of 100 ng/mL. However, 200 and 400 ng/mL of LPS caused obvious cytotoxicity to Raw264.7 cells. Therefore, 100 ng/mL LPS was chosen to stimulate Raw264.7 cells for 24 h and to establish an *in vitro* inflammation model.

The cytotoxicity of PA stock solution, CCMTS-P and PA/CCMTS-P were tested in Raw264.7 cells after 24 h treatment. The results showed that there was change in the viability of RAW264.7 cells when the concentration of the PA stock solution was below 0.4 mg/mL. Similarly, PA/CCMTS-P containing below 0.4 mg/mL of PA stock solution did not induce cytotoxicity to Raw264.7 cells (Figures 2B, C). As shown in Figure 2D, 1:225 diluted solution of CCMTS-P had obvious cytotoxic effects on Raw264.7 cells. Hence, 0.2 mg/mL PA, PA/CCMTS-P, and 1:450 dilution of CCMTS-P were selected for cellular uptake and inflammatory cytokines tests.

### CCMTS markedly improves drug uptake and permeability by targeting macrophages

**FIGURE 3:**
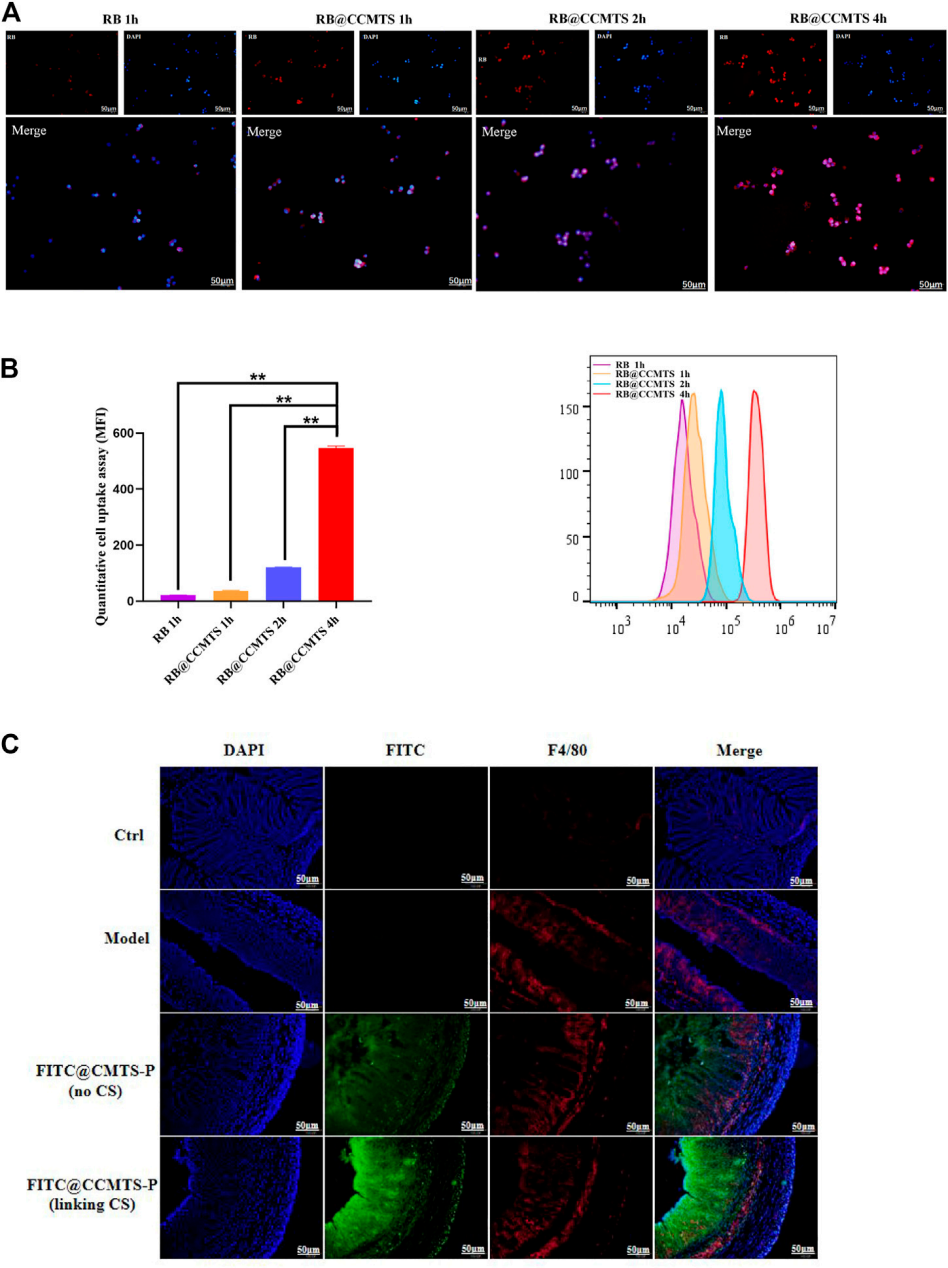
CCMTS-P markedly improves drug uptake and permeability by targeting macrophages *in vivo* and *in vitro*. **(A)** Fluorescence images of the distribution of CCMTS-P in Raw264.7 cells at 1, 2, 4 h. Scale bars, 50 μm **(B)** measurement of the quantitative cellular uptake of CCMTS-P by Raw264.7 cells at 1, 2, 4 h by flow cytometry. (*n* = 3 independent experiments) **(C)** permeation of free FITC, FITC@CMTS-P and FITC@CCMTS-P into the colon tissue. Scale bars, 50 μm. Data are shown as mean ± SD, **p < 0.01.

To investigate whether the higher anti-inflammatory effect of CCMTS was related to its ability to target of macrophages, RB was used to label CCMTS. As shown in Figure 3A, the fluorescence of free RB in Raw264.7 cells was weak and almost invisible, while the fluorescence signal in the CCMTS group was bright and intense. Cellular uptake tests *in vitro* showed that in comparison with the blank group, CCMTS was internalized more effectively by Raw264.7 cells after LPS stimulation. Meanwhile, the fluorescence intensity in Raw264.7 cells increased with longer incubation time, indicating that the uptake ability of CCMTS was time-dependent. After 4 h, the accumulation of RB labeled CCMTS in nucleus was significantly higher than that at 2, 1 h, which suggested the time-dependent uptake of CCMTS by Raw 264.7 cells through endocytosis. Similarly, the results of the flow cytometry histogram showed that the uptake of RB labeled CCMTS by Raw 264.7 cells was also time-dependent. As shown in Figure 3B, the flow intensity of CCMTS was significantly higher than that of the blank group, and the flow intensity was remarkably increased after 4 h (vs. 2 h, 1 h and blank group, *p* < 0.01).

To verify whether FITC labeled CS-functionalized CCMTS-P could specifically penetrate into macrophages in the colitis tissues, the frozen sections were immunostained with the F4/80 antibody. As shown in Figure 3C, the red fluorescence of the F4/80 antibody showed the location of macrophages and the green fluorescence of FITC showed CMTS-P and CCMTS-P. F4/80 was only rarely distributed in the colon tissue of control group, while in the other three groups, the fluorescence intensity was brighter and stronger. In the FITC@CCMTS-P group, FITC was highly overlapped with the red fluorescence signal of F4/80, and the fluorescence intensity was brighter than that of the FITC@CMTS-P group. These results indicated that FITC@CCMTS-P had better selectivity for colon macrophages than FITC@CMTS-P, mainly because of the ability of CS to specifically conjugate to the CD44 receptor on macrophages. The above results confirmed that CCMTS-P had good macrophage and colon targeting ability.

### PA/CCMTS-P significantly improves the symptoms in DSS-induced mouse model of UC

**FIGURE 4:**
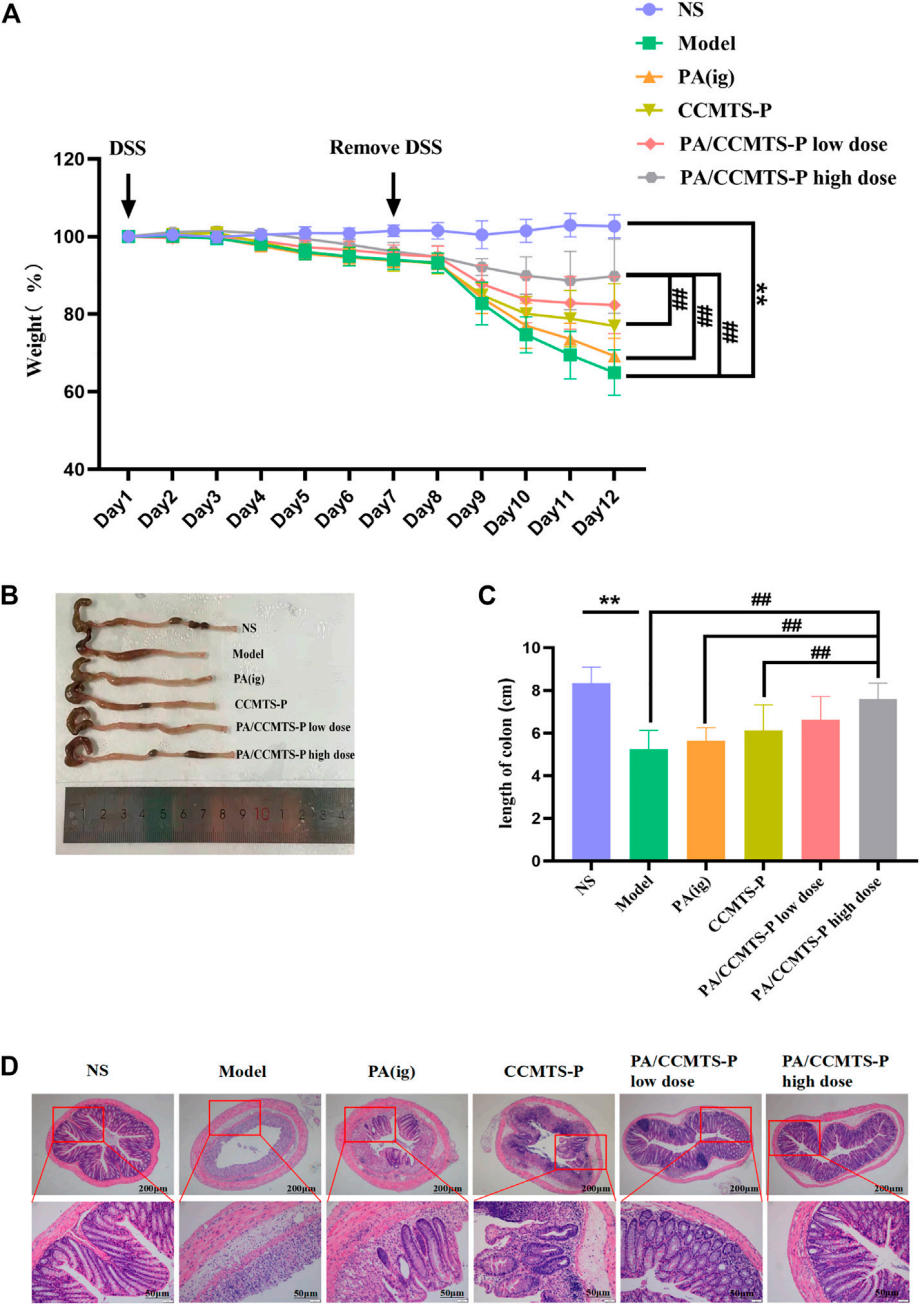
PA/CCMTS-P markedly ameliorates the pathological symptoms of DSS-induced UC mice. **(A)** The body weight change of mice in each group during the experiment from Day1 to Day 12. **(B)** Representative colon images at day 12. **(C)** Statistical graph of colon length in each group on Day 12 (*n* = 6). **(D)** Representative images of H and E staining of the colon tissue of mice in each group. Scale bars, 200 and 50 μm. Data are shown as mean ± SD (*n* = 6), **p < 0.01, #*p* < 0.05, ##*p* < 0.01.

Based on the role of DSS in UC induction, we explored the protective efficacy of PA/CCMTS-P in DSS-induced mouse model of UC. The body weight of the mice was recorded daily. As shown in Figure 4A, the body weight in the model group revealed a dramatic decrease after 7 days of 3% DSS drinking, while treatment with PA/CCMTS-P significantly improved the weight loss caused by DSS. Meanwhile, on day 12, compared with the PA (i.g.) and CCMTS-P groups, high dose of PA/CCMTS-P showed better results on weight loss. Colon length reflects the severity of inflammatory infiltration in the colon and is also an important indicator for evaluating the efficacy of UC treatment strategies. The images of the collected colon samples showed that the colon length of the model group was significantly shortened. While in the PA/CCMTS-P high dose group, after 5 days of therapy, colonic shortening was effectively alleviated and there was no obvious difference in the colon length between the NS and PA/CCMTS-P high dose group (Figures 4B, C).

H and E staining reflects the inflammatory injury and epithelial integrity of colon tissues. As shown in Figure 4D, the model group mice showed severe gland disappearance, inflammatory cell infiltration, obvious edema and extensive necrosis of the intestinal crypt. However, the administration of PA/CCMTS-P effectively protected the structure of the crypts with better epithelial integrity, fewer ulcers and milder inflammatory cell infiltration. Moreover, the PA/CCMTS-P high dose group had the most obvious protective effects on colon epithelial cells relative to the PA (i.g.), CCMTS-P and PA/CCMTS-P low dose group. These results showed that PA/CCMTS-P alleviated inflammation and accelerated wound healing after DSS injury in mice.

### PA/CCMTS-P protects against injury to the intestinal mucosal barrier

**FIGURE 5:**
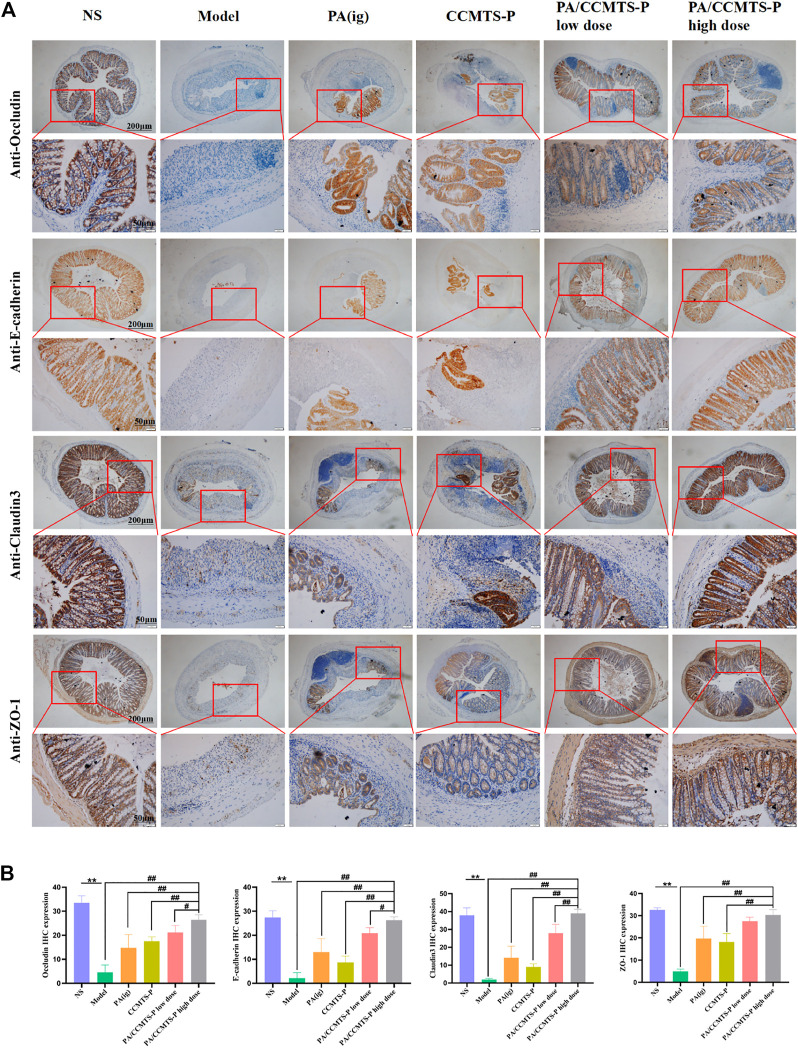
The protective effect of PA/CCMTS-P on intestinal mucosal barrier in DSS-induced UC model. **(A)** IHC evaluation of the four representative TJ and AJ proteins Occludin, E-cadherin, Claudin-3 and ZO-1. Scale bars, 200 and 50 μm. **(B)** Histochemical positive area statistics for Occludin, E-cadherin, Claudin-3 and ZO-1 (*n* = 3). Data are shown as means ± SD (*n* = 3),**p < 0.01; #*p* < 0.05, ##*p* < 0.01.

The tight connectivity of intestinal tight junctions (TJs) and adherent junctions (AJs) proteins are important for the formation of an intact intestinal immune barrier. An intact intestinal mucosal barrier prevents harmful substances such as bacteria or toxins from entering other tissues or blood circulation through the intestinal mucosa. Destruction of the intestinal barrier function is the typical clinical manifestation of UC (Mehandru and Colombel, 2021). ZO-1 is one of the representative tight junction proteins connected with the cytoskeleton protein, and Occludin plays a vital role in the formation and regulation of TJs and AJs. Claudin-3 plays a major role in TJ-specific obliteration of the intercellular space. In addition, E-Cadherin belongs to a superfamily of transmembrane glycoproteins, which mediates calcium-dependent cell-cell adhesion and plays a critical role in normal tissue development (Yin Y et al., 2022). These four indexes reflect the integrity and permeability of the intestinal barrier. The immunohistochemistry results are shown in Figures 5A, B. Compared with the NS group, the expression levels of ZO-1, Occludin, Claudin-3 and E-Cadherin were significantly decreased in the model group, indicating that the intestinal barrier was seriously damaged. While PA/CCMTS-P (low and high dose) dramatically increased the expression levels of these four proteins in the DSS-induced UC colon, and the effect of high dose PA/CCMTS-P group was higher than that in the PA (i.g.) and CCMTS-P treatment groups, indicating that PA/CCMTS-P treatment was effective at protecting the intestinal mucosal barrier.

### PA/CCMTS-P effectively alleviates inflammation both *in vivo* and *in vitro*

**FIGURE 6:**
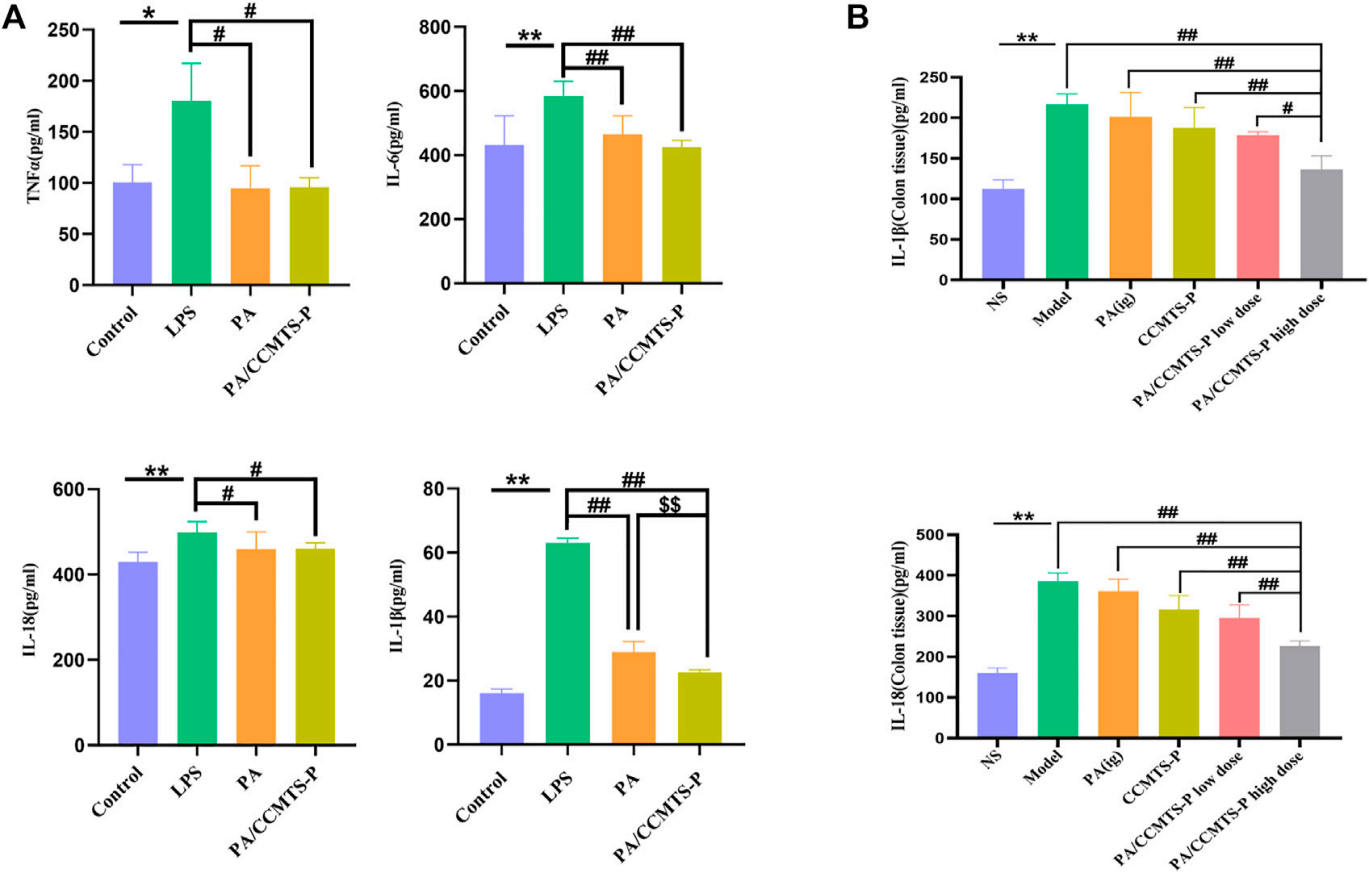
PA/CCMTS-P effectively alleviates inflammation both *in vivo* and *in vitro.* **(A)** The expression levels of inflammatory cytokines TNFα, IL-6, IL-18, and IL-1β in the supernatant of RAW264.7 were measured by ELISA after stimulation with LPS with or without PA and PA/CCMTS-P for 24 h. Data are shown as mean ± SD (*n* = 5), *p < 0.05, **p < 0.01; #*p* < 0.05, ##*p* < 0.01; $$*p* < 0.01. **(B)** The expression levels of inflammatory cytokines IL-1β and IL-18 in the colon tissues of DSS-induced UC mice were measured by ELISA. Data are shown as mean ± SD (*n* = 3), **p < 0.01; #*p* < 0.05, ##*p* < 0.01.

Inflammation is a typical manifestation of UC and the imbalanced secretion of inflammatory factors is closely associated with the occurrence and development of UC. To demonstrate the anti-inflammatory effects of PA and PA/CCMTS-P, LPS stimulated Raw264.7 cells were used as inflammation model *in vitro*. As shown in Figure 6A, after stimulation with LPS for 24 h, the levels of proinflammatory cytokines such as TNFα, IL-6, IL-18 and IL-1β were obviously increased, while PA and PA/CCMTS-P had good regulatory effects on these proinflammatory factors. Both PA and PA/CCMTS-P significantly reduced the levels of TNFα, IL-6, IL-18, and IL-1β. Additionally, PA/CCMTS-P was more effective than PA at decreasing the level of IL-1β (*p* < 0.01). The above results showed that PA/CCMTS-P had excellent anti-inflammatory effects *in vitro*.

Since inflammatory cytokine expression plays an essential role in the pathogenesis of UC and the severity of UC is directly related to the number of inflammatory cytokines, we studied the anti-inflammatory effects of PA/CCMTS-P in the DSS-induced UC model. As shown in Figure 6B, in the Model group, IL-1β and IL-18 were significantly increased compared to the NS group (*p* < 0.01), while the levels of IL-1β and IL-18 in the DSS-induced UC model were significantly reduced after PA/CCMTS-P treatment. Furthermore, PA/CCMTS-P showed higher anti-inflammatory effects as compared with the PA (ig) and CCMTS-P groups (*p* < 0.01) and this was dose-dependent. These results were consistent with the anti-inflammatory findings *in vitro*.

### PA/CCMTS-P attenuates colitis by modulating RIP1/RIP3/MLKL signaling mediated necroptosis and activation of the NLRP3 inflammasome

**FIGURE 7:**
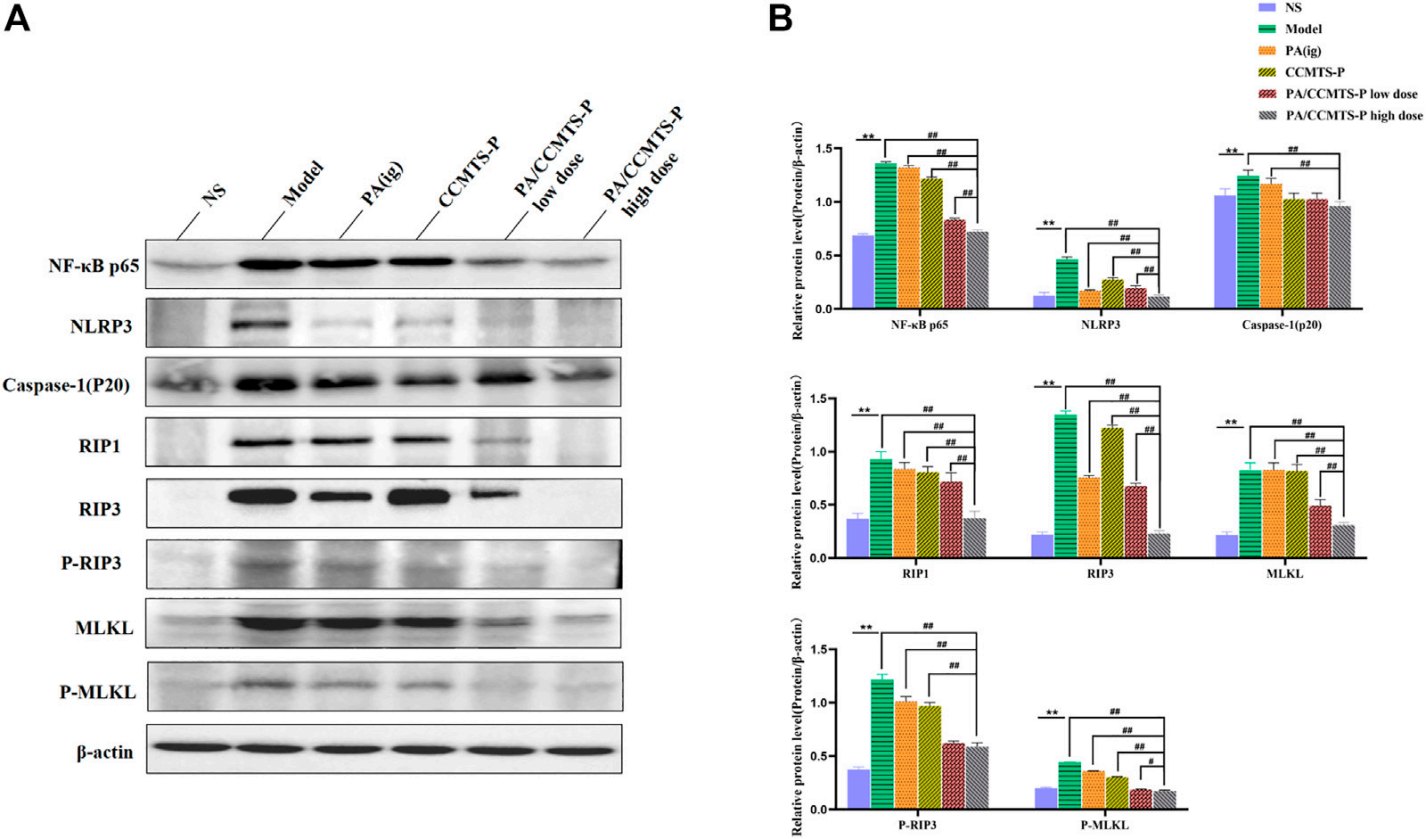
PA/CCMTS-P attenuates DSS-induced UC by modulating RIP1/RIP3/MLKL signaling mediated necroptosis and the activation of the NLRP3 inflammasome. **(A)** Western blotting analysis showed that PA/CCMTS-P inhibited the RIP1/RIP3/MLKL necroptosis pathway as well as NF-κB and NLRP3 inflammasome activation in DSS-induced UC mice model. **(B)** Quantitative analyses of western blotting. Data are shown as mean ± SD (*n* = 3 independent experiments), **p < 0.01; #*p* < 0.05, ##*p* < 0.01.

In order to determine whether PA/CCMTS-P was involved in the modulation of necroptosis-mediated inflammasome signaling, we measured the expression of several key proteins in colon tissues through Western blotting. As shown in Figures 7A, B, significant differences were observed in expression of NF-κB p65, NLRP3, Caspase-1 (p20), RIP1, RIP3, and MLKL between the NS and Model group. (*p* < 0.01). However, PA/CCMTS-P treatment remarkably decreased the expression of NF-κB p65, NLRP3, Caspase-1 (p20), RIP1, RIP3 and MLKL (*p* < 0.01). Compared with the PA (i.g.) and CCMTS-P groups, PA/CCMTS-P treatment reduced the expression of NF-κB p65, NLRP3, Caspase-1 (p20), RIP1, RIP3, and MLKL more effectively, and this effect was dose-dependent. MLKL is a key player in necroptotic signaling, and its phosphorylation is a specific marker of necroptosis activation (Wang H et al., 2014). As shown in Figures 7A, B, the level of RIP3 and MLKL phosphorylation in the model group were significantly increased in comparison to the control group. Additionally, compared with the Model, PA (ig) and CCMTS-P groups, the expression level of phosphorylated RIP3 and MLKL were significantly reduced in the PA/CCMTS-P group in a dose-dependent manner. All these results revealed that PA/CCMTS-P strongly inhibited RIP1/RIP3/MLKL mediated necroptosis as well as NF-κB and NLRP3 inflammasome activation in UC mice model, which further alleviated inflammation in the DSS-induced UC model.

## Discussion

Immune dysregulation is the main pathogenesis associated with UC, including abnormal levels of inflammatory factors and leukocyte recruitment, defects in epithelial barrier and colonic microflora (Nishida et al., 2018). However, the clinical needs of UC are still largely unmet, and UC continues to be a global challenge. Protecting the intestinal barrier, inhibiting excessive cell necrosis and inflammation may be effective approaches for treating UC.

Recently, colon-targeted drug delivery systems (DDSs) have received increasing attention as potential therapeutic approach for treating UC. For example, magnolol was loaded into core−shell zein-composed nanoparticles with chondroitin sulfate coating, which was then embedded into hydrogel microspheres through electrospraying technology. The product showed significant macrophage-targeting, enhanced colon epithelial cellular uptake capacity, prolonged colon retention and excellent anti-inflammatory effects in UC patients. The above CS-functionalized medicine was demonstrated to specifically target the colonic macrophages through CD44 receptor activation in the colitis site (Wang et al., 2021). In our study, CCMTS-P showed great ability to improve drug uptake and permeability by targeting macrophages as well. Besides, CS modification increased the selectivity for colon macrophages. The specificity of CCMTS-P for colon macrophages and its colon permeability make it a promising candidate for anti-UC therapy.

TISG has solution-gel transition property, which rapidly transforms into gel state and adheres to the drug site with temperature change, can be administered in various ways, including luminal administration (Permana et al., 2021), mucosal administration (Maddiboyina et al., 2021), transdermal administration (Khan et al., 2019), and subcutaneous injection (Yu et al., 2017). Kangfuxin liquid (KFX) is a patented Chinese medicine of PA, which have been used for UC treatment orally or rectally (Ma et al., 2018; Xue NN et al., 2020). However, the low availability after being exposed to the distal colon and short retention time limit the therapeutic effects of KFX in UC patients. Therefore, an *in situ* hydrogel containing KFX was designed by using temperature-sensitive P-407 as the material for rectal administration, which improved the therapeutic effect of KFX liquid in DSS-induced UC model (Xue P et al., 2020). In our design, P407-CHO was chemically cross-linked with CMCS-CS by Schiff base reaction, and mixed with excipients such as Poloxamer 188 and *ε*-polylysine to encapsulate PA to produce PA/CCMTS-P. After enema administration, PA/CCMTS-P transformed into a high-strength hydrogel state in the rectum due to phase transformation, which prolonged the retention time of PA in the colon and improved intracellular drug delivery mediated by macrophages within the inflammatory site.

DSS-induced UC mouse model is the most widely used, replicable, and classic animal model of UC. The typical manifestations in these mice include weight loss, diarrhea and hematochezia, and the appearance of congestion, edema, erosion, and even intestinal mucosal ulcers and colon length shortening (Chassaing et al., 2014). In our study, after 5 days of PA/CCMTS-P therapy, the body weight and colon length shortening were obviously improved, as well as the pathological changes including inflammatory cell infiltration, mucosal ulcers and extensive necrosis of the intestinal crypts were partially healed. All these findings indicate the therapeutic efficacy of PA/CCMTS-P in DSS-induced colitis model.

Intestinal epithelial cells, goblet cells, paneth cells, and the mucus layer form the first intestinal barrier. TJs and AJs are vital components of the intestinal mucosal barrier. The proteins occludin, claudin3, and ZO-1, are representatives of the TJs, which form a barrier at the top of the adjacent epithelial cell membrane to prevent the paracellular transport of intercellular molecules. The role of AJs proteins such as E-cadherin is to interact with tight junction proteins and form adhesions between adjacent epithelial cells to close the intestinal barrier (Mehandru and Colombel, 2021). Once the homeostasis of the intestinal epithelial barrier is perturbed by endogenous and exogenous factors, the increased permeability of the intestinal mucosa further triggers the immune system, thus aggravating mucosal inflammation (Mehandru and Colombel, 2021). Medicines focusing on the restoration of the homeostasis of the intestinal epithelial barrier are considered as the primary therapeutic modalities for treating UC patients (Cholapranee et al., 2017). In our study, we found that the intestinal mucosal barrier in DSS-induced UC mice was severely damaged, while PA/CCMTS-P was effective at restoring the damaged mucous layer, upregulating the protein expression of TJs and AJs and rebuilding the integrity of the intestinal epithelial barrier in the inflamed colon tissues. In addition, the therapeutic effect of rectal administration of PA/CCMTS-P was better than that in the PA (i.g.) and CCMTS-P groups.

Previous studies demonstrated that PA could ameliorate DSS-induced UC by regulating immune responses and modulating several signaling pathways, such as the PI3K/AKT/NF-κB and Keap1/Nrf-2 signaling pathways (Ma et al., 2018; Ni et al., 2022). In UC rats, both Th1/Th2 and Th17/Treg were up-regulated due to the remarkably increased effects on pro-inflammatory factors. Besides, activated NF-κB signaling also promoted the secretion of pro-inflammatory cytokines, including ILs, IFN-γ, and TNF-α (Sadar et al., 2016). These findings from the above studies were consistent with our results. PA/CCMTS-P showed excellent anti-inflammatory effects both *in vitro* and *in vivo*, which obviously reduced the secretion of TNF-α, IL-6, IL-1β, and IL-18 after LPS stimulation in Raw264.7 cells, and the expression of IL-1β and IL-18 at the colitis site *in vivo*.

Previously, some studies reported that necroptosis was involved in promoting inflammation and damage to the intestinal epithelium cells (Wu et al., 2019; Wu et al., 2021; Xiao et al., 2021). Necroptosis, also known as programmed necrosis, is characterized by cell swelling, mitochondrial dysfunction, plasma membrane permeabilization, and the release of cytoplasmic content into the extracellular space. Necroptosis is mainly mediated by the activation of RIP1, RIP3 and P-MLKL. Moreover, phosphorylated MLKL translocates to the cell membrane and destroys the integrity of the cell membrane, which is also the primary executor of necroptosis (Giampietri et al., 2014). Damage-associated molecular patterns (DAMPs) are released from the dying necrotized cells, which are then recognized by PRRs (Pattern recognition receptors), leading to the subsequent activation of innate immunity such as the Toll and NLR (Nod-like receptor) signaling pathways to evoke a series of inflammatory responses. NLRP3 is an important NLR, which forms the inflammasome by activating caspase-1 and by regulating the processing of pro-IL-1β, and pro-IL-18 and the secretion of mature and active cytokines IL-1β and IL-18 (Rayamajhi and Miao, 2014). The activation of NLRP3 is involved in the progression of UC and its inhibition is known to ameliorate DSS-induced colitis (Wu et al., 2020). In this study, the protein expression levels of RIP1, RIP3, and MLKL in mice with DSS-induced colitis were significantly higher than in the control mice, and the rectal administration of PA/CCMTS-P could significantly reverse this phenotype. The expression levels of p-RIP3 and p-MLKL also showed the same trend. Additionally, PA/CCMTS-P attenuated DSS-induced increase in the expression of NLRP3 and caspase-1 (p20) in the colon tissues, which are known to be involved in the formation of NLRP3 inflammasome, as well as reduced the expression levels of colonic IL-1β and IL-18. Furthermore, the downstream NF-κB p65 inflammatory signaling pathway was also influenced by PA/CCMTS-P. All the above findings confirmed the therapeutic efficacy of PA/CCMTS-P in UC and that it was mediated by the inhibition of the RIP1/RIP3/MLKL signaling pathway and activation of the NLRP3 inflammasome. Compared with the other treatment groups, PA/CCMTS-P (high dose) had the most optimal therapeutic effects. However, further studies are needed to evaluate the long-term effects of PA/CCMTS-P in animal models with chronic UC and unravel the underlying molecular mechanisms.

## Conclusion

In conclusion, the prepared thermosensitive enema *in situ* hydrogels based on chondroitin sulfate modified carboxymethyl chitosan-Poloxamer 407 carrying PA (PA/CCMTS-P) could effectively alleviate colonic injury and inhibit the inflammatory response in mice with DSS-induced ulcerative colitis. Besides, *in vitro* experiments revealed that CCMTS-P markedly improved drug uptake and permeability by targeting macrophages. PA/CCMTS-P showed obvious effects on inhibiting the release of inflammatory cytokines both *in vivo* and *in vitro*. The underlying protective mechanism was found to be associated with the regulation of necroptosis mediated by the RIP1/RIP3/MLKL signaling pathway, which suggested that PA/CCMTS-P enemas could serve as a promising therapeutic strategy for ulcerative colitis.

## Data Availability

The raw data supporting the conclusion of this article will be made available by the authors, without undue reservation.
